# Prognostic impact of serum and tissue MMP-9 in non-small cell lung cancer: a systematic review and meta-analysis

**DOI:** 10.18632/oncotarget.7607

**Published:** 2016-02-23

**Authors:** Liang Gong, Dan Wu, Jianding Zou, Jianqiang Chen, Liangyu Chen, Yun Chen, Chao Ni, Hongjun Yuan

**Affiliations:** ^1^ Department of Otorhinolaryngology, Affiliated Cixi Hospital of Wenzhou Medical University, Cixi 315300, China; ^2^ Department of Thoracic surgery, Affiliated Cixi Hospital of Wenzhou Medical University, Cixi 315300, China; ^3^ Department of Oncology, Zhejiang Provincial People's Hospital, Hangzhou 310004, China; ^4^ Department of General Surgery, Zhejiang Provincial People's Hospital, Hangzhou 310004, China

**Keywords:** matrix metalloprotease-9, non-small cell lung cancer, immunohistochemistry, ELISA

## Abstract

Matrix metalloproteinases-9 (MMP-9) was one of the most important enzyme to breakdown extracellular matrix, aim to clarify the prognostic value of MMP-9 in non-small cell lung cancer (NSCLC), we investigated the serum MMP-9 of NSCLC patients and performed a meta-analysis of the published literature. The expression and activity of serum MMP-9 were assessed by ELISA and gelatin zymography in 163 NSCLC patients. Moreover, 26 studies were included in meta-analysis by searching Medline and ISI Web of Knowledge. Our own data revealed high activity but not expression of MMP-9 significantly correlated with advanced T category and positive metastasis. In contrast, the meta-analysis revealed that increased MMP-9 level indicate high T category (RR = 0.83, 95% CI: 0.73-0.94), tumor stage (RR = 0.72, 95% CI: 0.63-0.82) and poor OS (5-year overall survival, RR = 1.32, 95% CI: 1.19-1.48). Moreover, stratified analysis based on sample types found that high MMP-9 expression in tissue specimen but not serum was significant correlated with advanced T category (RR = 0.81, 95% CI: 0.72-0.92), tumor stage (RR = 0.69, 95% CI: 0.60-0.80) and poor 5-year OS (1.33, 95% CI: 1.18-1.50). In conclusion, the activity of MMP-9 was positively correlated with advanced T category and distant metastasis. Moreover, the meta-analysis revealed that overexpression of MMP-9 in tissue but not in serum was a risk factor of advanced T category, tumor stage and poor outcome.

## INTRODUCTION

Despite improvements in diagnostic and therapeutic strategies, non-small cell lung cancer (NSCLC) still remains the most lethal malignancy worldwide. The 5-year survival rate is only 15% overall in patients and 50-60% in resectable patients [1]. These results also revealed invasion and metastasis as the main causes of recurrence and death in lung cancer.

Numerous studies have found that the degradation of the extracellular matrix (ECM) induced by certain enzymes plays a central role in tumor metastasis. The matrix metalloproteinases (MMPs) family comprises 24 zinc dependent endopeptidases with various enzymatic activities to break down ECM. MMP-9 is part of the MMPs family and is the most important component in cancer tissue remodeling, able to catalyze types V, VII, IX, X, and IV collagen, elastin, fibrin, fibrinogen and plasminogen to facilitate malignant cell invasion and metastasis [2]. Moreover, the inhibition of MMP-9 was reported to attenuate the invasion of tumor cells [3, 4], which indicated its potential as both a cancer marker and therapeutic target. Based on the above evidence, the increased expression of MMP-9 should correlate with poor prognosis and advanced tumor stage, theoretically. However, MMP-9 has been evaluated in various human specimens, such as serum and tissue, and several studies found that high MMP-9 expression was not correlated with unfavorable clinicopathological features or poor prognosis, especially when evaluated in patient serum, which may be due to a small sample size in individual studies [5–8].

In this study, we examined MMP-9 expression and activity in the serum of 163 NSCLC patients by enzyme-linked immunosorbent assay (ELISA) and gelatin zymography, which provided the largest current sample size. Moreover, because MMP-9 was most frequently assessed in tumor specimens, we also performed a systematic review of published research to clarify the prognostic significance of MMP-9 in tissue and serum, and other clinicopathological features were also examined in this study.

## RESULTS

### Patient characteristics

A total of 163 specimens of NSCLC patients (from April 2006 to March 2011) from Wenzhou Medical college affiliated Cixi People's hospital with complete follow-up were retrospectively studied. All of the patients' pathological and demographic characteristics are shown in Supplementary Table S1. The predominant histological type of NSCLC was adenocarcinoma (79 patients, 48.5%), followed by squamous cell carcinoma (76 patients, 46.6%) and large cell carcinoma (8 patients, 4.9%). At the time of diagnosis, 71 (43.6%) patients had early stage disease (stage I/II), 37 (22.7%) patients had locally advanced tumors (stage III) and 55 (33.7%) patients had metastatic disease (stage IV).

### Expression and activity of MMP-9 in serum

The mean serum level of MMP-9 in NSCLC patients was 67.08±39.57 ng/ml, and the association between MMP-9 level and activity with pathological parameters was shown in Table 1. Although we found that patients with a higher MMP-9 concentration are more inclined to have an advanced clinicopathological feature (N and M stage) and worse prognosis, none of these factors reached statistical significance (Table 1, Supplementary Figure S3). Alternatively, the activity of MMP-9 was significantly correlated with advanced T category and positive metastasis (Table 1, Supplementary Figure S1).

**Table 1:** Correlations between patients' backgrounds with the expression and activity of MMP-9 in 163 NSCLC patients

Characteristics	No. of Cases with plasma MMP-9 concentration of			No. of Cases with plasma MMP-9 activity of		
	>67.08ng/ml	≤67.08ng/ml	p	>1.1	≤1.1	p
**Gender**						
**Male**	45	37	0.390	52	30	0.426
**Female**	39	42		46	35	
**T stage**						
**I**	34	52	0.066	33	53	0.0006
**II-IV**	41	35		49	27	
**Lymph node metastasis**						
**Yes**	47	44	0.700	49	42	0.507
**No**	35	37		35	37	
**Distant metastasis**						
**Yes**	30	25	0.199	33	22	0.002
**No**	31	41		23	49	
**Tumor stage**						
**I/II**	31	40	0.092	33	38	0.101
**III/IV**	50	32		49	33	
**3-year OS**						
**Dead**	27	24	0.403	30	21	0.079
**Live**	42	50		40	52	

### Search results

**Figure 1:**
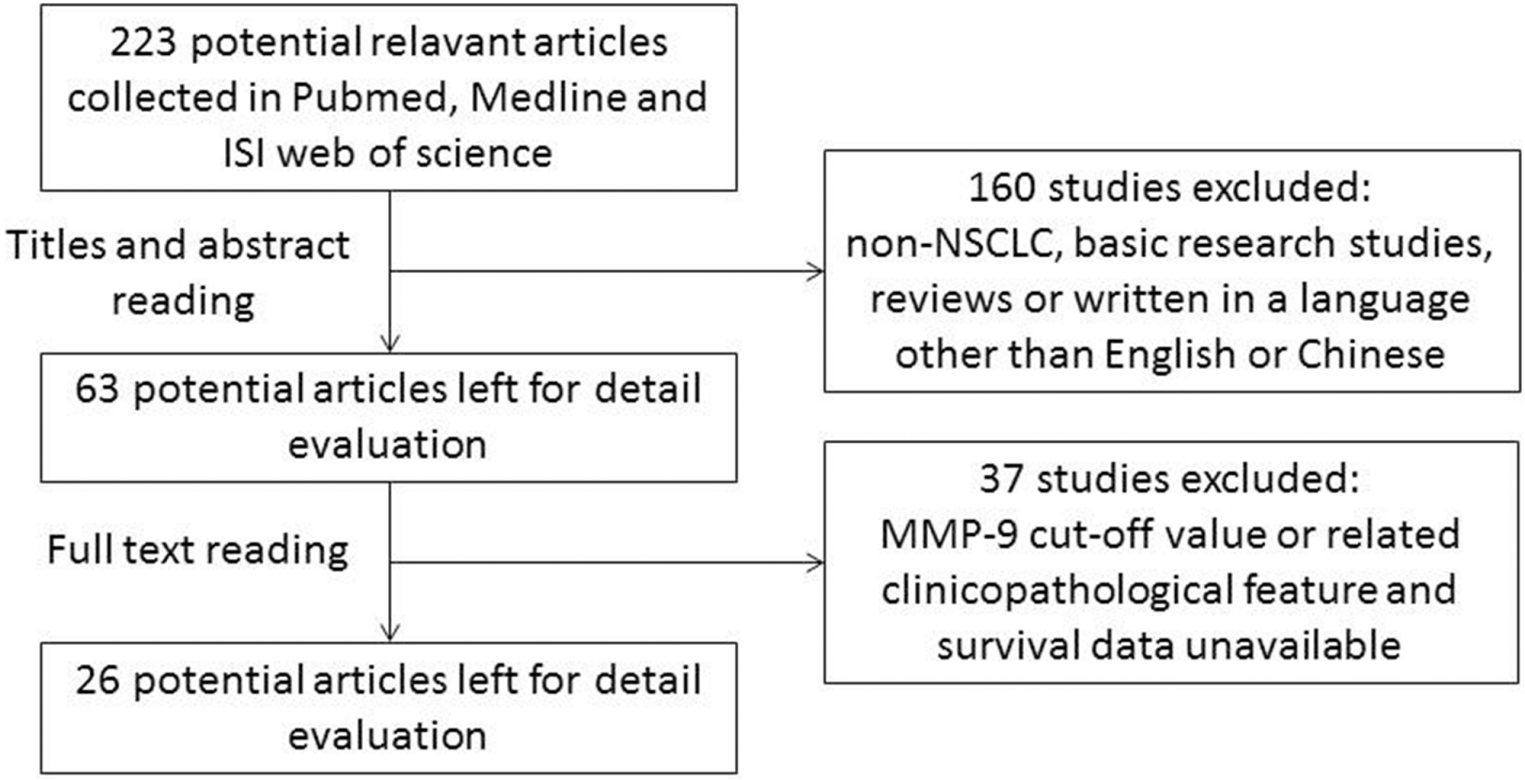
Flow chart for selection of studies

Because several studies indicated that MMP-9 level could be applied as a prognostic marker in NSCLC patients, either in serum or tissue specimens, here we performed a meta-analysis with comprehensive research of the published literature. Initially, 223 publications were retrieved by our primary research. Thereafter, 160 studies were excluded based on abstracts due to either a focus on non-NSCLC, basic research studies, reviews or written in a language other than English or Chinese. Then, the full-texts of the remaining 63 studies were carefully reviewed. Thirty-seven studies were excluded because they did not provide a defined cut-off of MMP-9 level or the detailed information about MMP-9 and clinicopathological features or survival rate was unavailable. Although we tried to contact all of the corresponding authors by e-mail to fill the data table, we were unable contact the authors within 6 weeks. Finally, including data presented here by our group, 27 studies were identified as eligible for this meta-analysis (Figure 1).

### Characteristics of eligible studies

Except for the current study, a total of 26 published studies from 1999 to 2015 including 2637 NSCLC patients were enrolled in this meta-analysis (Table 2), which included 15 retrospective studies [1, 6, 7, 9–20], 9 observational studies [8, 21–28] and 2 prospective studies [5, 29]. Nineteen studies comprising 1964 subjects were conducted in Asia and 8 studies comprising 836 subjects were conducted in Western countries (Europe or USA). MMP-9 level was evaluated in the serum in 5 studies by ELISA, 21 studies used immunohistochemistry to detect MMP-9 and 1 study used a PCR method to assess the MMP-9 level in tumor tissue homogenate. None of the included patients received neo-adjuvant chemo- or radiotherapy pre-surgery. Furthermore, because the cut-off value for MMP-9 varied among studies, we defined the MMP-9 high expression group with respect to the original works.

**Table 2:** Main characteristics of the included studies in this meta-analysis

No.	Author, year	Country	No. of patients	Gender (male/female)	Specimen, Method	T stage (1/2-4)	N stage (pos/neg)	Distant metastasis (pos/neg)	Tumor Stage (1,2/3,4)	DFS (relapse/free)	3-year OS (dead/alive)	5-year OS (dead/alive)	NOS score
1	Iizasa, 1999	Japan	73	H(24/21) L(9/19)	Serum, ELISA	NA	H(22/24); L(11/16)	H(33/37); L(0/3)	H(19/23); L(14/17)	NA	NA	NA	6
2	Cox, 2000	UK	169	H(59/59) L(29/22)	Tissue, IHC	H(76/76); L(12/5)	H(41/47); L(47/34)	NA	H(63/67); L(25/14)	NA	H(49/32); L(29/52)	H(59/22); L(45/36)	8
3	Zhao, 2000	China	62	H(31/9) L(19/4)	Tissue, IHC	H(16/7); L(34/5)	H(11/27); L(23/1)	NA	H(26/11); L(24/1)	NA	H(23/27); L(0/12)	H(33/17); L(1/11)	7
4	Shou, 2001	Japan	119	NA	Tissue, IHC	NA	NA	NA	NA	NA	H(31/44); L(7/29)	H(44/31); L(11/25)	8
5	Cao, 2003	China	40	NA	Tissue, IHC	NA	H(6/6); L(1/27)	NA	H(9/5); L(24/2)	NA	NA	NA	5
6	KAYA, 2003	Turkey	35	NA	Serum, ELISA	H(5/16); L(6/8)	H(16/5); L(12/2)	H(11/10); L(4/10)	H(3/8); L(2/12)	NA	NA	NA	5
7	Liang, 2003	China	65	NA	Tissue, IHC	H(15/32); L(8/10)	NA	H(32/15); L(5/13)	H(23/24); L(16/2)	NA	NA	NA	6
8	Sienel, 2003	Germany	143	H(22/88) L(4/29)	Tissue, IHC	H(10/64); L(16/53)	H(39/35); L(38/31)	NA	H(14/49); L(8/46)	NA	H(71/35); L(10/14)	H(91/15); L(11/13)	8
9	Wang, 2003	China	104	NA	Tissue, IHC	H(57/18); L(21/8)	H(43/7); L(35/19)	NA	H(46/2); L(18/1)	NA	NA	NA	6
10	Kim, 2005	USA	74	NA	Tissue, IHC	H(48/23); L(3/0)	H(42/19); L(9/4)	NA	NA	NA	H(9/42); L(6/17)	H(15/36); L(7/16)	8
11	Shimanuk, 2005	Japan	61	NA	Serum, ELISA	NA	NA	NA	NA	NA	H(20/10); L(19/12)	H(21/9); L(20/10)	6
12	Leinonen, 2006	Finland	197	NA	Tissue,IHC	H(31/21); L(80/61)	H(29/31); L(82/51)	NA	H(42/49); L(51/23)	H(51/62); L(37/47)	NA	H(78/35); L(58/26)	7
13	Wang, 2005	China	64	H(33/12) L(16/7)	Tissue, IHC	NA	H(26/4); L(23/15)	NA	H(36/19); L(13/0)	NA	NA	NA	6
14	Guo, 2007	China	87	NA	Tissue, IHC	NA	NA	H(34/15); L(21/17)	H(20/18); L(35/14)	H(37/18); L(16/16)	H(15/40); L(7/25)	H(28/27); L(12/20)	8
15	Iniesta, 2007	Spain	92	NA	Tissue, ELISA	NA	NA	NA	NA	H(24/23); L(31/14)	NA	NA	5
16	Chen, 2008	China	41	H(16/9) L(11/5)	Tissue, IHC	H(10/11); L(17/6)	H(10/11); L(17/3)	NA	H(13/12); L(14/2)	NA	H(24/3); L(3/11)	NA	7
17	Grossi, 2008	UK	87	NA	Tissue, IHC	NA	NA	NA	NA	NA	H(34/9); L(30/14)	H(37/6); L(32/12)	7
18	Lim, 2010	Korea	41	NA	Tissue, IHC	H(12/21); L(3/5)	H(4/17); L(11/9)	NA	H(4/15); L(11/10)	NA	NA	NA	5
19	Liu, 2010	China	95	H(51/28) L(11/5)	Tissue, IHC	H(7/10); L(55/23)	H(28/23); L(34/10)	H(56/33); L(6/0)	H(31/26); L(31/7)	NA	NA	NA	7
20	Shao, 2011	China	146	H(59/36) L(30/21)	Tissue, IHC	NA	NA	NA	NA	NA	H(25/64); L(4/53)	H(25/64); L(5/52)	8
21	Li, 2012	China	65	NA	Tissue, IHC	NA	H(17/9); L(17/22)	H(19/13); L(15/18)	H(23/12); L(11/19)	NA	NA	NA	6
22	Schveigert, 2013	Poland	20	NA	Tissue, PCR	NA	H(10/3); L(7/0)	NA	H(6/4); L(8/1)	NA	NA	NA	6
		Poland	19	NA	Serum, PCR	NA	H(9/4); L(6/1)	NA	H(7/3); L(9/0)	NA	NA	NA	
23	Tang, 2013	China	80	H(50/18) L(9/3)	Tissue, IHC	H(4/1); L(55/20)	H(56/16); L(3/5)	NA	H(15/13); L(44/8)	NA	NA	NA	5
24	Lee, 2015	Korea	473	H(100/179) L(61/77)	Tissue, IHC	H(33/75); L(128/181)	H(53/52); L(108/204)	NA	H(128/228); L(33/28)	H(58/103); L(54/202)	H(33/128); L(48/218)	H(54/107); L(69/187)	8
25	Liu, 2015	China	87	H(24/18) L(28/17)	Tissue, IHC	H(30/29); L(22/6)	H(20/23); L(32/12)	NA	H(13/20); L(39/15)	NA	NA	NA	6
26	Su, 2015	China	98	H(41/12) L(28/17)	Tissue, IHC	NA	H(23/14); L(46/15)	H(46/24); L(23/5)	H(27/15); L(42/14)	H(54/15); L(18/11)	H(50/19); L(14/15)	H(54/15); L(18/11)	8
27	Gong, 2015	China	163	H(49/33) L(46/37)	Serum, ELISA	H(52/35); L(41/35)	H(58/33); L(35/37)	H(35/20); L(58/50)	H(40/31); L(53/39)	NA	H(32/61); L(19/51)	NA	7

H: high expression; L: low expression; NA: not available; IHC: immunohistochemistry; NOS, Newcastle–Ottawa Quality Assessment Scale.

### Correlation of MMP-9 expression and clinicopathological parameters

**Figure 2:**
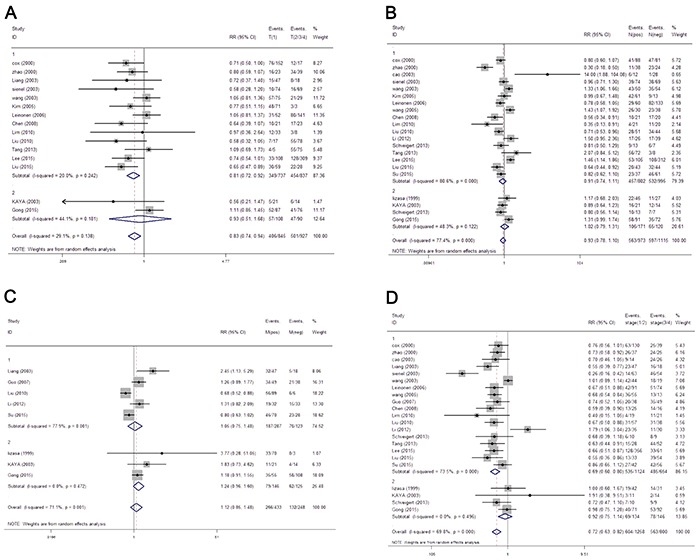
The forest plot of RRs was assessed for association between MMP-9 and clinicopathological features, including T category **A.** lymph node metastasis **B.** distant organ metastasis **C.** and tumor grade **D.** Each result was shown by the RR with 95% CIs (according to the fixed model).

The correlation between MMP-9 expression and overall gender, T category, N category, metastasis status and tumor stage was illustrated in Figure 2. Fifteen studies evaluated the association of MMP-9 expression and T category. The pooled RR was 0.83 (95% CI: 0.73-0.94) without heterogeneity (I^2^ = 29.1%, p = 0.138). While the subgroup analysis was performed based on sample types (tissue or serum), we only found an increased risk of advanced T category with high MMP-9 expression in tissue specimens and not in serum (tissue RR = 0.81, 95% CI: 0.72-0.92; serum RR = 0.93, 95% CI: 0.51-1.68). Moreover, we evaluated the correlation between tumor stage and MMP-9 expression in twenty-two eligible studies, and the overall results indicate a positive association between high MMP-9 expression and advanced tumor stage (RR = 0.72, 95% CI: 0.63-0.82) with heterogeneity (I^2^ = 69.8%, p = 0.000). To explain the heterogeneity, we performed a subgroup analysis and found that high MMP-9 expression was also associated with advanced tumor stage only in tissue specimens and not serum (tissue RR = 0.69, 95% CI: 0.60-0.80, serum RR = 0.92, 95% CI: 0.75-1.14).

However, the results did not reveal a significant relationship between overall MMP-9 expression with either lymph node or distant organ metastasis (N category RR = 0.93, 95%CI: 0.78-1.10; M category RR = 1.12, 95%CI: 0.85-1.48). Similar results were also found in the sub-group analysis (N category tissue RR = 0.91, 95% CI: 0.74-1.11, serum RR = 1.02, 95% CI: 0.79-1.31; M category tissue RR = 1.05, 95% CI: 0.85-1.48, serum RR = 1.24, 95% CI: 0.96-1.60). Gender was also not a parameter that correlated with MMP-9 expression either in overall or subgroup analysis based on sample types (RR = 1.0, 95% CI: 0.91-1.09, Supplementary Figure S2).

### Impact of MMP-9 expression on overall survival and disease-free survival of NSCLC

**Figure 3:**
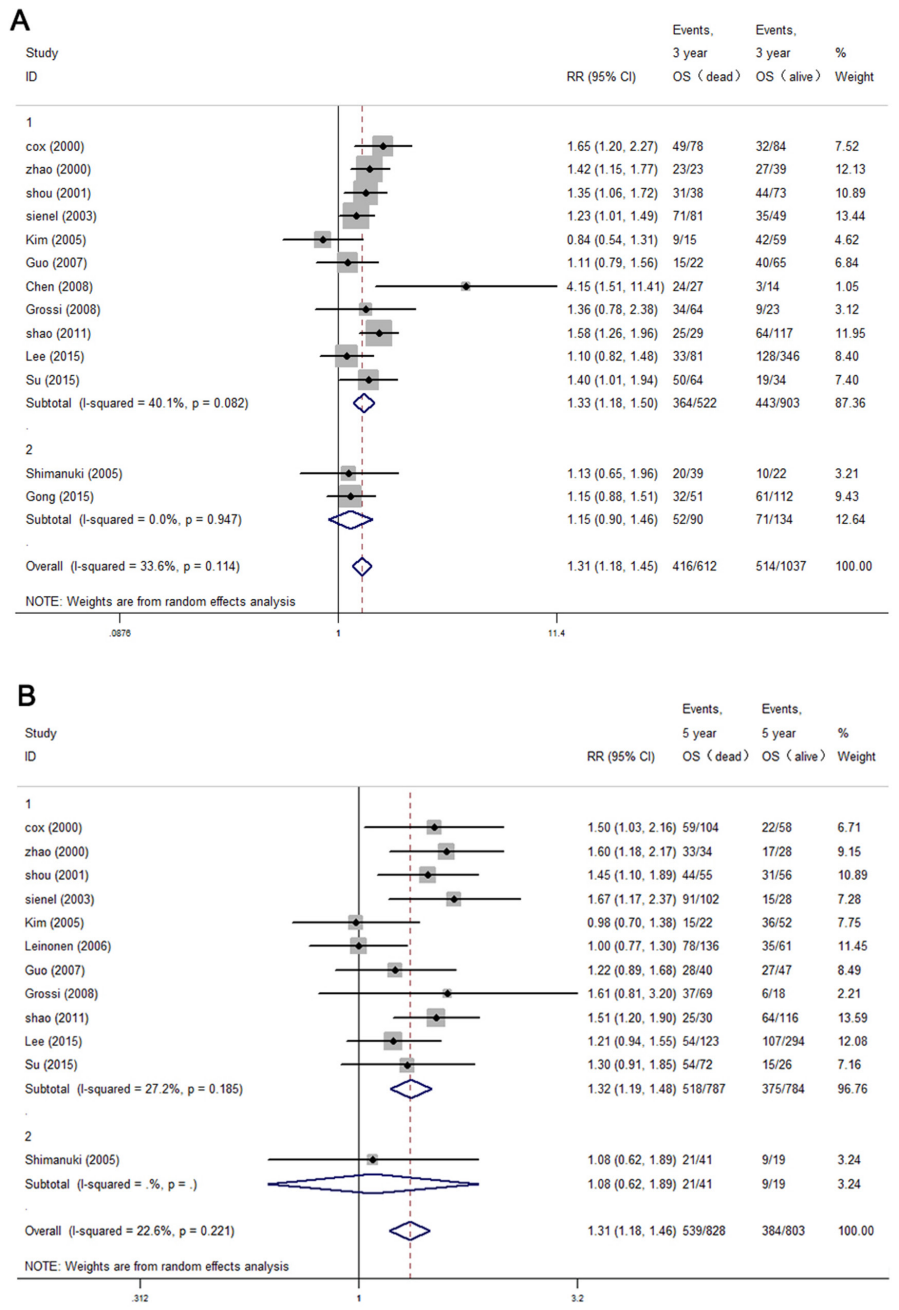
The forest plot of RRs was assessed for association between MMP-9 expression and 3-year overall survival **A.** 5-year overall survival **B.** Each result was shown by the RR with 95% CIs (according to the fixed model or random model).

The 5-year DFS data were extracted from 5 articles all studying tissue specimens (Figure 3), and the pooled analysis found that an increased expression of MMP-9 did not correlate with disease progression (pooled RR = 1.15, 95% CI: 0.89-1.48). Then, we assessed whether the MMP-9 level was associated with OS (Figure 3). The 3-year OS rate of the MMP-9 high and low groups was 55.3% (514/930) and 62.9% (1037/1649), respectively, while the 5-year OS was 41.6% (382/923) and 49.2% (803/1631), respectively. Our analysis revealed that MMP-9 significantly indicated poor overall survival in both 3-year and 5-year OS (pooled 3-year RR = 1.31, 95% CI: 1.18-1.45; pooled 5-year RR = 1.32, 95% CI: 1.19-1.48). Because there was only one study with serum samples that provided information on the 5-year OS rate, the stratified analysis was only performed for the 3-year OS. It was interesting that the subgroup analysis again found that high MMP-9 expression in tissue but not serum indicated a worse prognosis (tissue group RR = 1.33, 95% CI: 1.18-1.50; serum group RR = 1.15, 95% CI: 0.90-1.46).

### Publication bias

Begg's and Egger's tests were both performed to determine the publication bias based on clinicopathological and survival parameters. The funnel plots were almost symmetrical and did not indicate any significant biases among the studies.

## DISCUSSION

Here we present a meta-analysis based on a large pool of studies (including 16 retrospective studies, 9 observational studies and 2 prospective studies) that evaluated the clinical relevance and prognostic value of MMP-9 in NSCLC patients. One systematic review regarding MMP-9 in NSCLC was published in 2012 and concluded that MMP-9 is associated with poor prognosis [30]. However, because of the limited information, the previous meta-analysis was confined to studies with immunohistochemistry assay to detect the MMP-9 expression in the tissue. Because the significance of the MMP-9 level in NSCLC still remains controversial, we constructed this meta-analysis and tried to illuminate this issue.

MMP-9 is secreted by various cells in an inactivated form as a 10 kDa propeptide, which could be activated by other MMPs or tissue plasminogen activator (tPA)-plasmin system, due to its biological function of cleaving gelatin, which promotes malignant cell motility [3, 4], MMP-9 was regarded as an important prognostic marker and therapeutic target of cancer in many studies. The biology of MMP-9 was comprehensively reviewed by Vandooren et al. [2]. In brief, basic studies have revealed that a high expression of MMP-9 is positively associated with the aggressiveness of malignant cells in solid tumors. Except for its well-known function of degrading the extracellular matrix, in vivo studies in MMP-9 deficient mice found that tumor metastasis was enhanced by implanting MMP-9 expression in the bone marrow, which could promote angiogenesis to facilitate cancer cell migration. Additionally, MMP-9 could be derived from stromal cells, such as inflammatory cells and fibroblasts [31, 32]. Furthermore, although a soluble enzyme, MMP-9 could also bind to CD44 to activate the TGF-β pathway to enhance angiogenesis and epithelial to mesenchymal transition [33, 34], and it could also down-regulate the expression of E-cadherin as a transcriptional factor [35].

Although all of the above mentioned evidence strongly supports the fact that MMP-9 could potentiate cancer aggressiveness, there still remains controversy among the clinical observational studies. In our study, 163 serum samples of NSCLC patients were collected and the circulating MMP-9 expression was assessed. Our retrospective analysis did not find any significant correlation between total serum MMP-9 expression and clinicopathological features or survival. Because the activity of MMP-9 could not be detected by ELISA, a gelatin zymography assay was performed, and high activity of MMP-9 was a risk factor of advanced tumor stage and distant metastasis, which is concordant with El-Badrawy et al.'s report [36]. In addition to our retrospective analysis, we also conducted a meta-analysis to address the prognostic value of MMP-9 in NSCLC; however, neither of the included studies determined its activity in tissue or serum. Our systematic analysis revealed that MMP-9 expression in tumor tissue was positively related to T category, tumor stage, and 3-year and 5-year OS rate while a high level of MMP-9 in the serum was not associated with any of these parameters.

The increment of circulating MMP-9 was released by tumor cells or stromal cells, and its activity could be downregulated by many extracellular factors, such as MMP-1, 3, 7, 10, 26, trypsin-2 and neutrophil elastase [37], among them, the tissue inhibitors of metalloproteinases 1 (TIMP1) is the most important endogenous inhibitor against MMP-9. TIMP1 belongs to a family of proteins that naturally inhibit MMPs, which consists of four members (TIMP1, TIMP2, TIMP3, and TIMP4). In fact, several studies revealed that TIMP1 not only blocks the cleavage effect of MMP-9 extracellularly but also inhibits the membrane-protein shedding process and cell signal regulatory effect of MMP-9 [38–40]. Although the anti-MMP-9 behavior led TIMP1 to be considered as a tumor suppress gene initially, later, researchers found that TIMP1 also functions independently of MMP-9 to promote tumor growth and inhibit apoptosis [41]. TIMP1 expression was increased in solid tumors, such as lung cancer, breast cancer and colorectal cancer, and was acknowledged as a risk factor of poor outcome [42–44]. Its high expression not only impedes the activity of MMP-9 but also potentiates the aggressiveness of malignant cells. Based on our own data and meta-analysis here, the activity of serum MMP-9 was associated with tumor stage and metastasis status, but its total expression in serum was not related to clinicopathological parameters or survival. Therefore, the activity of circulating MMP-9 may not be proportional to the amount detected by ELISA. Weng et al. found that MMP-9 activity was reduced in lung cancer tissue [45], which could also be proof of this corollary.

This meta-analysis is subject to a few limitations. First, almost 75% of patients were from studies in Asia, which may have induced bias; second, the number of studies with serum samples is relatively small, and the survival information was unavailable for most of them; third, although subgroup studies in stratified analysis applied a consistent method to determine the expression of MMP-9, the criteria still varied among individual studies, which may be attributed to different antibodies and the influence of pathologists; fourth, our meta-analysis is based on published literature, and thus, some information was missed and individual patient data were unattainable, which could decrease the accuracy of the results. In spite of these limitations, our meta-analysis result revealed that high MMP-9 expression in tumor tissue but not serum could be an implication of advanced tumor stage and poor prognosis of NSCLC. It should be prudent to detect serum MMP-9 expression as a prognostic factor in the future, and a parallel study with a large number of subjects is needed in the future to compare the clinical significance of total expression of tissue MMP-9 and the activity of MMP-9 both in tumor tissue and serum.

## MATERIALS AND METHODS

### Subjects and specimen collection

A total of 163 patients with NSCLC, including 76 squamous cell carcinoma, 79 adenocarcinoma and 8 large cell carcinoma, of the Wenzhou Medical College Affiliated Cixi People's Hospital (Zhejiang province, China) were included in this study. For all of the patients, the diagnosis was confirmed by histological examination of either biopsy or cytologic specimen. Informed consent was obtained from each patient, and also permitted by the ethic committee of Wenzhou Medical College Affiliated Cixi People's Hospital. Venous blood samples were collected from all of the patients via routine venipuncture. The blood samples were obtained using an EDTA anti-coagulant tube, immediately centrifuged and the serum was stored at-80°C. The survival information was available in 143 patients, while the rest were lost to follow-up.

### Measurement of MMP-9 by enzyme-linked immunosorbent assay (ELISA)

The expression of MMP-9 in the serum was measured using a human MMP-9 ELISA kit (R&D Systems, Abingdon, UK) by following the manufacturer's instruction strictly, and all of the samples were measured in triplicate.

### Measurement of MMP-9 by gelatin zymography

MMP-9 activity in serum was detected by gelatin zymography as described previously [36]. Briefly, the protein concentration of the serum samples was determined using the Bradford protein assay. Then, 20 mg of total protein was electrophoresed on a 10% sodium dodecyl sulfate polyacrylamide gel at room temperature for 1 h. After electrophoresis, the gels were treated with Triton X-100 for 30 minutes at room temperature followed by incubation in a reaction buffer containing 50 mM Tris (pH 7.4), 5 mM CaCl2, and 150 mM NaCl for 24 hours at 37°C, then stained with Coomassie blue. The activity of MMP-9 was considered a band at 78 kDa, and MMP-9 activity was analyzed by NIH image software (v1.62). The activity of MMP-9 in the samples was determined as the percentage ratio between the intensity of the gel analytic bands in the samples in relation to the standard MMP-9 (92 kDa) (NBP1-99195-Novus Biologicals).

### Literature search strategy

We performed a comprehensive literature search of Medline and ISI Web of Knowledge up to November of 2015 with regard to the prognostic significance of MMP-9 in NSCLC. Search items included “MMP-9” or “matrix metallopreoteinases-9” and “NSCLC” or “non-small cell lung cancer.” The titles and abstracts of publications identified by the search were examined manually to exclude reviews, letters, basic research and other irrelevant studies.

### Eligibility criteria

The studies in this meta-analysis included both prospective or retrospective studies, and the studies were selected if they met the following criteria: (a) focused on NSCLC and more than 20 patients, (b) they defined MMP-9 expression in the tumor specimen or serum, (c) clear analysis of correlations between MMP-9 expression and clinicopathological features or survival outcomes (overall or disease free survival), and (d) the studies were published in English or Chinese.

To control for the quality of this meta-analysis, all of the enrolled studies were examined with seven key points provided by the Dutch Cochrane Centre: (a) a clear definition of study population and country of origin, (b) a clear definition of the type of carcinoma, (c) a clear definition of the study design, (d) a clear definition of the outcome assessment, (e) a clear definition of the cut-off of MMP-9 expression, (f) a clear definition of the method of MMP-9 assessment and (g) a sufficient time for follow-up.

### Data extraction

All of the data were extracted independently by two reviewers. The following data were extracted using a standardized method: author's name, publication year, country, number of patients, detection method, type of specimen, cut-off value for MMP-9, T category, N category, distant metastasis, DFS rate and OS rate. For studies that provided OS or PFS with a Kaplan-Meier curve, GetData Graph Digitizer 2.24 software (http://getdata-graph-digitizer.com/) was used to digitize and extract the data from the Kaplan-Meier curves. We also tried our best to collect the missing information by emailing the corresponding author.

### Statistical analysis

For the data provided by our group, the results are presented as the mean±standard error of the mean, and all of the data were analyzed by the Mann–Whitney rank sum test between groups with GraphPad Prism 6.0. For the meta-analysis, the extracted data were analyzed with guidelines proposed by the Meta-Analysis of Observational Studies in Epidemiology group. Relative risk (RR) with a 95% confidence interval (95% CI) was determined with Review Manager 4.2. The heterogeneity among studies was measured by Q and *I^2^* tests. A fixed or random model was used depending on the heterogeneity analysis. The potential for publication bias was also evaluated using the Begg rank correlation method and the Egger weighted regression method (Stata11.0 software). A *P* value >0.05 was considered statistically significant. All *P* values are two-tailed.

## SUPPLEMENTARY FIGURES AND TABLES


