# Supplementary material for: Prognostic impact of serum and tissue MMP-9 in non-small cell lung cancer: a systematic review and meta-analysis
Source: Oncotarget. 2016 Feb 23;7(14):18458–68. doi: 10.18632/oncotarget.7607 (PMC4951301; doi:10.18632/oncotarget.7607)
Supplement: Supplementary file 1 [file oncotarget-07-18458-s001.pdf]

SUPPLEMENTARY FIGURES AND TABLE

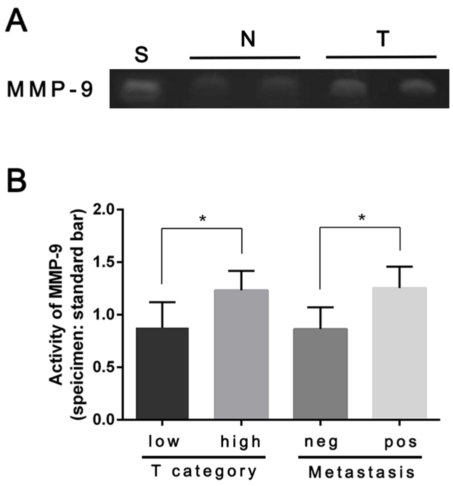

Supplementary Figure S1: MMP-9 activity is serum samples. A. representative gelatin zymography indicating the activities of MMP-9 in healthy person (N) and NSCLC patients (T), Lane S: Standard active MMP-9 (78 kDa); **B.** histogram revealed NSCLC patients with advanced T category and distant metastasis have significant higher MMP-9 activity in serum.* p<0.05.

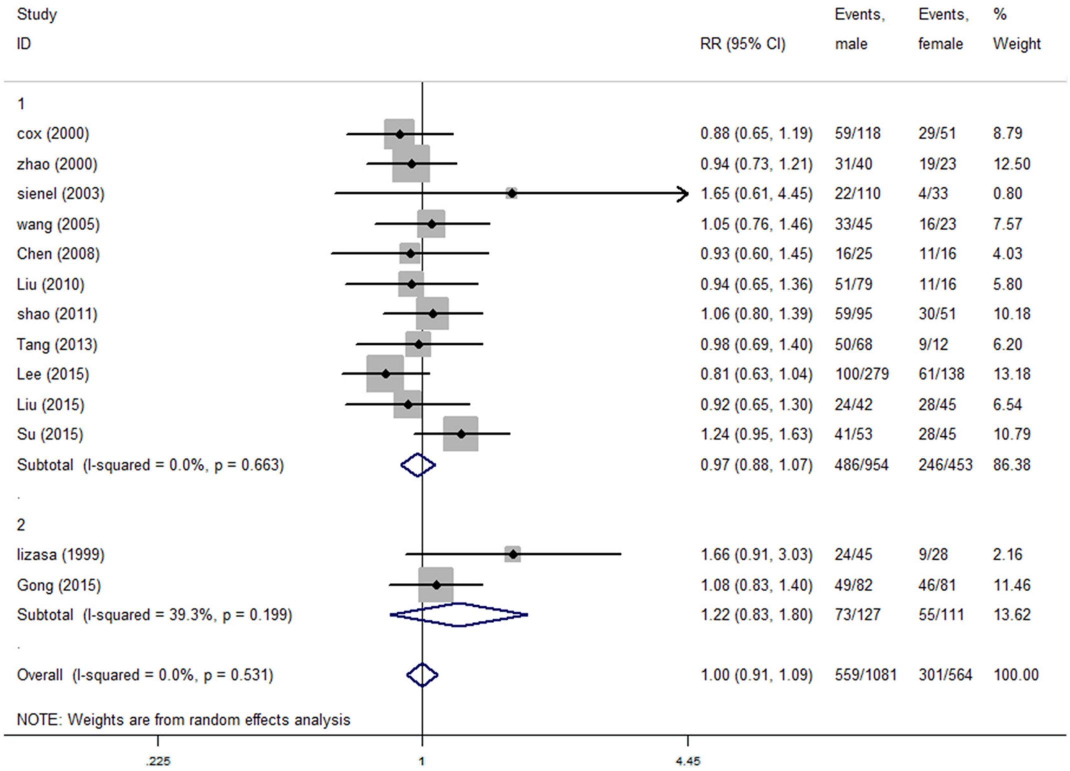

Supplementary Figure S2: The forest plot of RRs was assessed for association between MMP-9 expression and gender A. disease-free survival **B.**

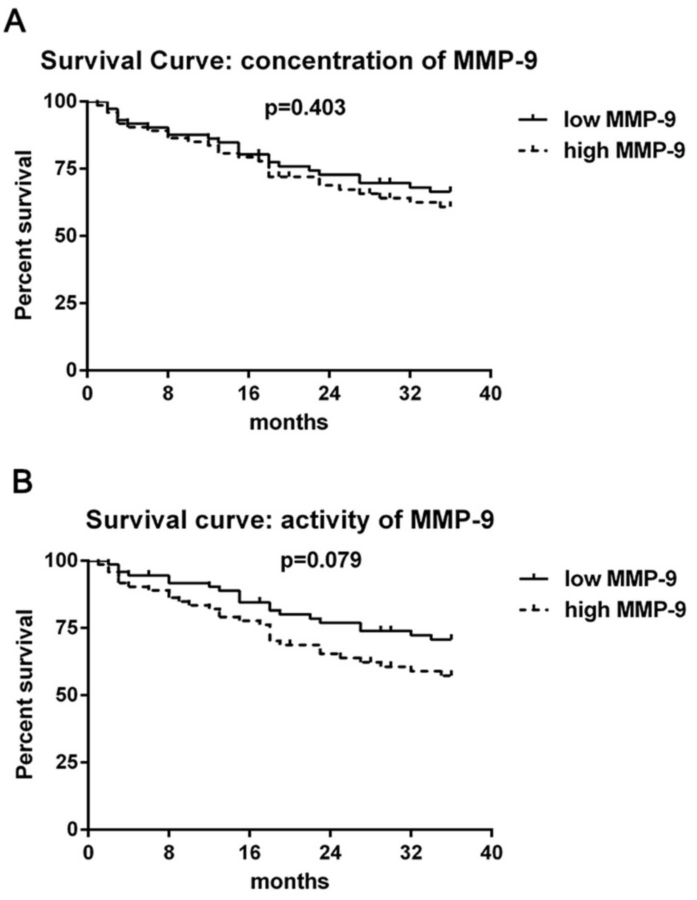

Supplementary Figure S3: A. The Kaplan-Meier Curve of low/high MMP concentration; **B.** The Kaplan-Meier Curve of low/high MMP activity.

Supplementary Table S1: Demographic features of 163 NSCLC patients

Clinicopathological features	Patients, n (%)
Gender	
Male	82 (50.3)
Female	81 (49.7)
Age,years	
>60	69 (42.3)
≤60	94 (57.7)
T stage	
I	87 (53.4)
II-IV	76 (46.6)
Lymph node metastasis	
Yes	91 (55.8)
No	72 (44.2)
Distant metastasis	
Yes	55 (33.7)
No	108 (66.3)
Tumor stage	
I	43 (26.4)
II	28 (17.2)
III	37 (22.7)
IV	55 (33.7)
Histological type(NSCLC)	
Adenocarcinoma	79 (48.5)
Squamous cell carcinoma	76 (46.6)
Large cell carcinoma	8 (4.9)
3-year OS	
Dead	51 (31.3)
Live	92 (68.7)
Lost	20 (12.3)
